# Respiratory microbes present in the nasopharynx of children hospitalised with suspected pulmonary tuberculosis in Cape Town, South Africa

**DOI:** 10.1186/s12879-016-1934-z

**Published:** 2016-10-24

**Authors:** Felix S. Dube, Mamadou Kaba, F. J. Lourens Robberts, Lemese Ah Tow, Sugnet Lubbe, Heather J. Zar, Mark P. Nicol

**Affiliations:** 1 Division of Medical Microbiology, Department of Pathology, Faculty of Health Sciences, University of Cape Town, Cape Town, South Africa; 2 Faculty of Health Sciences, Institute for Infectious Diseases and Molecular Medicine, University of Cape Town, Cape Town, South Africa; 3 Department of Statistical Sciences, Faculty of Science, University of Cape Town, Cape Town, South Africa; 4 Department of Paediatrics and Child Health, Red Cross War Memorial Children’s Hospital, University of Cape Town, Cape Town, South Africa; 5 SAMRC Unit on Child and Adolescent Health, University of Cape Town, Cape Town, South Africa; 6 National Health Laboratory Service, Groote Schuur Hospital, Cape Town, South Africa

**Keywords:** Infection, Nasopharynx, Microbiota, *Mycobacterium tuberculosis*, Tuberculosis, Respiratory microbes

## Abstract

**Background:**

Lower respiratory tract infection in children is increasingly thought to be polymicrobial in origin. Children with symptoms suggestive of pulmonary tuberculosis (PTB) may have tuberculosis, other respiratory tract infections or co-infection with *Mycobacterium tuberculosis* and other pathogens. We aimed to identify the presence of potential respiratory pathogens in nasopharyngeal (NP) samples from children with suspected PTB.

**Method:**

NP samples collected from consecutive children presenting with suspected PTB at Red Cross Children’s Hospital (Cape Town, South Africa) were tested by multiplex real-time RT-PCR. Mycobacterial liquid culture and Xpert MTB/RIF was performed on 2 induced sputa obtained from each participant. Children were categorised as definite-TB (culture or qPCR [Xpert MTB/RIF] confirmed), unlikely-TB (improvement of symptoms without TB treatment on follow-up) and unconfirmed-TB (all other children).

**Results:**

Amongst 214 children with a median age of 36 months (interquartile range, [IQR] 19–66 months), 34 (16 %) had definite-TB, 86 (40 %) had unconfirmed-TB and 94 (44 %) were classified as unlikely-TB. *Moraxella catarrhalis* (64 %), *Streptococcus pneumoniae* (42 %), *Haemophilus influenzae spp* (29 %) and *Staphylococcus aureus* (22 %) were the most common bacteria detected in NP samples. Other bacteria detected included *Mycoplasma pneumoniae* (9 %), *Bordetella pertussis* (7 %) and *Chlamydophila pneumoniae* (4 %). The most common viruses detected included metapneumovirus (19 %), rhinovirus (15 %), influenza virus C (9 %), adenovirus (7 %), cytomegalovirus (7 %) and coronavirus O43 (5.6 %). Both bacteria and viruses were detected in 73, 55 and 56 % of the definite, unconfirmed and unlikely-TB groups, respectively. There were no significant differences in the distribution of respiratory microbes between children with and without TB. Using quadratic discriminant analysis, human metapneumovirus, *C. pneumoniae*, coronavirus 043, influenza virus C virus, rhinovirus and cytomegalovirus best discriminated children with definite-TB from the other groups of children.

**Conclusions:**

A broad range of potential respiratory pathogens was detected in children with suspected TB. There was no clear association between TB categorisation and detection of a specific pathogen. Further work is needed to explore potential pathogen interactions and their role in the pathogenesis of PTB.

**Electronic supplementary material:**

The online version of this article (doi:10.1186/s12879-016-1934-z) contains supplementary material, which is available to authorized users.

## Background

Lower respiratory tract infection (LRTI) is a leading cause of mortality and morbidity in children under five years, accounting for approximately 1 million deaths in 2013 globally [1]. Pulmonary tuberculosis (PTB) is an important cause of LRTI and may present as acute or chronic disease [2]. TB is increasingly recognised as a primary cause or as a comorbid condition in children hospitalized with pneumonia, particularly in settings endemic for human immunodeficiency virus (HIV) and TB [3].

Diagnosis of PTB in children is largely made based on clinical and radiological features, which may be non-specific. Therefore PTB cannot be easily differentiated from other causes of acute or chronic LRTI [4]. Recent studies have reported detection of polymicrobial infections in children with LRTI [5]. Further, PTB has increasingly been reported in children presenting with acute pneumonia; culture confirmed TB was reported in 8 % of South African children hospitalized with acute pneumonia with no difference by HIV status [6]. A recent meta-analysis confirmed *Mycobacterium tuberculosis* in 7.5 % of childhood pneumonia cases in TB endemic areas [3].

There are limited published data on the role of other respiratory pathogens amongst patients suspected to have TB [7]. In Africa, only one study, conducted in Botswana, has addressed this question amongst adult PTB suspects. This study reported microbiologically confirmed TB in 118/229 (52 %); *Mycoplasma pneumoniae* infection in 36/218 (17 %) and *Pneumocystis jirovecii* infection in 4/141 (3 %) of PTB suspects [7]. Co-infection with two or more pathogens was documented in 25 % of patients [7].

In our studies of South African children hospitalised with suspected PTB, 16 % of children hospitalised with suspected PTB had microbiologically-confirmed PTB, 48 % were classified as unconfirmed-PTB and 37 % were classified as unlikely-PTB (children in whom TB was excluded and who improved in the absence of TB therapy) [8]. Approximately half of the children were treated for PTB, including all children with definite PTB and most with unconfirmed PTB [8]. These data raise several questions around potential co-infections including the extent to which these may contribute to disease or severity in PTB, the aetiology of LRTI amongst children with symptoms suggestive PTB and the role of other potential respiratory pathogens in those without TB.

We have recently shown [5] that specific pathogens (including *Bordetella pertussis*, influenza virus, respiratory syncyntial virus [RSV], adenovirus, parainfluenzavirus, bocavirus) are detected significantly more frequently from the nasopharynx (NP) of children with pneumonia than age-matched controls, and so are likely to play an important role in pneumonia aetiology. We therefore conducted a preliminary study to investigate the diversity of potential respiratory pathogens in the NP of children hospitalised with suspected PTB.

## Methods

### Study population and TB testing

The population studied in this study has been previously described [8]. Briefly, we enrolled children under 15 years of age suspected of PTB presenting (between July 2011 and May 2012) at Red Cross War Memorial Children’s Hospital (RCH), a specialist referral paediatric hospital in Cape Town, South Africa. Verbal assent was obtained from children older than seven (7) years of age and informed consent was obtained from a parent or legal guardian for all children.

Suspected TB was defined as having history of a cough and one of the following: i) a household contact with TB within the previous three months, ii) loss of weight or failure to gain weight in the previous three months, iii) a positive tuberculin skin test (TST) to purified protein derivative (PPD; 2TU, PPD RT23, Staten Serum Institute, Denmark, Copenhagen), or iv) a chest radiograph suggestive of PTB. A positive skin test was defined as 5 mm or more of transverse induration in children with HIV infection or 10 mm or more in children without HIV infection [9]. Children were excluded if the child was on TB treatment or TB prophylaxis for more than 72 h, and if they could not be followed-up (not resident in Cape Town). All patients with laboratory confirmed TB and those diagnosed with TB based on clinical and radiological criteria were referred for TB therapy at a local clinic in accordance with South African National Guidelines [10]. Children were followed up at 1 and 3 months to assess response to therapy or improvement without TB treatment. All children received care in the public health system that includes free expanded programme on immunisation (EPI) for diphtheria, pertussis (whooping cough), and tetanus (DPT), *Haemophilus influenza* type b, and *Streptococcus pneumoniae* (7-valent pneumococcal conjugate vaccine [PCV] from 2009, replaced with 13-valent PCV in 2011).

### Sample collection

Two paired induced sputa and a nasopharyngeal (NP) swab specimens were collected from each child and transported to the laboratory within 2 h of collection. NP swabs were obtained before sputum induction using nylon flocked swabs (Copan Italia, Brescia, Italy) by trained study staff [11]. Each NP swab was immediately placed into 1.5 ml PrimeStore® transport and stabilization medium (PrimeStore® MTM, Longhorn Vaccines and Diagnostics, San Antonio, TX) and stored at −80 °C within 2 h of collection until further batch processing. Samples were randomly selected from a convenience subset of 214 children, over a 1-year period for testing of NP specimens for other microbes.

As the volume obtained on induced sputum specimens was small, the entire specimen was required for detection of *M tuberculosis*, for optimal management and as this study was nested within a broader study investigating better diagnostics for TB in children [11]. We did not want to compromise this primary aim and only NP samples were available for study of other respiratory pathogens.

### Diagnosis of PTB

Induced sputum specimens were submitted to the National Health Laboratory Services (NHLS) Medical Microbiology Laboratory at Groote Schuur Hospital (Cape Town, South Africa) for mycobacterial liquid culture (BACTEC MGIT, Becton Dickinson Microbiology Systems, Cockeysville, MD) and nucleic acid amplification testing (Xpert MTB/RIF, Cepheid, Sunnyvale, CA).

Children were categorised as ‘definite-TB’ (i.e. culture or Xpert MTB/RIF positive for *M. tuberculosis*), ‘unlikely TB’ (i.e. no clinical diagnosis of TB with improvement on follow-up without TB treatment) and ‘unconfirmed TB’ (all others) [12].

### Multiplex PCR testing of nasopharyngeal samples

NP swabs were thawed at room temperature (22 °C) and vortexed for 15 s. Thereafter, 400 μl of each sample was transferred to a ZR BashingBeadsTM Lysis tube (Zymo Research Corp., Irvine, CA) and subjected to mechanical lysis on a Tissuelyzer LT (Qiagen, Hilden, Germany) [13]. The lysed samples were then centrifuged at 10,000 × g for 1 min to pellet all cellular debris. Aliquots of 250 μl of the supernatant were transferred to a 2 ml sterile tube (Sarstedt, Nümbrecht, Germany) and 4 μl of an exogenous internal control (Equine arteritis virus) was added to each sample prior to automated total nucleic acid extraction on the QIAsymphony SP instrument using the QIAsymphony® Virus/Bacteria mini kit (Qiagen, Hilden, Germany). Total nucleic acid was eluted in 60 μl elution buffer and stored at -80 °C until further processing.

Nucleic acid amplification was performed using the FTD Resp33 kit according to the manufacturer’s instructions (Fast-track Diagnostics, Luxembourg). The assay comprises eight multiplex real-time PCR reactions for the detection of nucleic acid targets (Additional file 1: Table S1).

Results were interpreted according to manufacturer’s instructions using the FTD resp33 Analyser, an in-house JAVA based program (available at http://www.gematics.com/analyser.html).

### Statistical analysis

Exploratory statistics were performed using STATA software (Stata Corporation, College Station, TX), whilst the openly available statistical environment R, version 3.1.1 [14] was used for more detailed analyses. Pearson’s Chi-squared test was used to compare the occurrence of each microbe between children with definite TB and unlikely TB with Yates’ continuity correction. Permutation tests were used to determine which microbe pairs were statistically concurrent. Briefly, for each pair of microbes, *X* and *Y,* the observed number of concurrences (*m*) was counted. The null hypothesis was that there was no relationship between microbes *X* and *Y* and that the co-occurrences were purely random. The null hypothesis was tested by generating random permutations of the occurrences of *X* and *Y*. The number of concurrences under these random conditions was then counted, *m*_*1*_. By repeating the permutation process 10 000 times, 10 000 random concurrences were obtained as follows: *m*_*1*_, *m*_*2*_,*…*, *m*_*10000*_. The shape of the null-distribution, the distribution of co-occurrence counts under purely random conditions, was estimated from the 10 000 observed values of the permutation test. The achieved significance level (ASL) was computed as the number of permuted co-occurrences that were equal to or greater than the observed number of concurrence (tail probability under the null-distribution). This can be interpreted as a non-parametric *p*-value [15].

Linear Discriminant Analysis (LDA) and Quadratic Discriminant Analysis (QDA) [16] were used to optimally discriminate respiratory microbes occurring in relation to TB status. The LDA is visually represented in a Canonical Variate Analysis (CVA) biplot and the QDA in a QDA biplot [16]. The visualizations provide information on how the different TB groups overlap and to what extent the detection of microbes differs between the groups. For all the tests, a *p*-value less than 0.05 was used as the limit of statistical significance.

### Ethical considerations

The Human Research Ethics Committee (**HREC 045/2008**) of the Faculty of Health Sciences, University of Cape Town, South Africa approved this study.

## Results

Table 1 summarises the baseline characteristics of the 214 children included in this study. The median age of the study participants was 36 months (interquartile range, [IQR] 19–66 months). Children with definite-TB were older (*p* = 0.003) than children with unconfirmed TB or unlikely TB. Thirty-four (16 %) of the 214 children had culture confirmed TB, while 86/214 (40 %) had unconfirmed-TB and 94/214 (44 %) were categorised as unlikely TB. Only 13 % (27/214) of children were HIV-infected, with similar HIV prevalence by TB category. Immunization records were available for 162 out of 214 (76 %) children included in this study. Among children for whom data on immunization profile was available, the vaccination status was up to date in 78 % of children, while among the remaining 22 % (36/162), at least one scheduled immunization was missing. None of the risk factors considered in Table 1 was associated with the occurrence of any microbes pertussis even after adjusting for potential confounders (Additional file 1: Table S3).

**Table 1:** Baseline characteristics of the children in the study by TB category

Characteristics	Total (*n* = 214)	Definite-TB (*n* = 34)	Unconfirmed-TB (*n* = 86)	Unlikely-TB (*n* = 94)	*p*-value
Age in months, median (IQR)	36 (19–66)	56 (23–109)	34 (15–63)	32 (17–63)	0.003
Sex: female, n (%)	109 (51)	14 (41)	45 (52)	50 (53)	0.03
HIV infection, n (%)	27 (13)	7 (21)	13 (15)	7 (7)	0.14
HIV WHO clinical staging, n (%)					
Stage 1	0	0	0	0	-
Stage 2	0	0	0	0	-
Stage 3	21 (10)	5 (15)	10 (12)	6 (6)	0.36
Stage 4	6 (3)	2 (6)	3 (35)	1 (1)	0.48
Smoking: yes, n (%)	29 (14)	5 (15)	12 (14)	12 (13)	0.56
Night sweats: yes, n (%)	118 (55)	15 (44)	57 (66)	46 (49)	0.61
Fever: yes, n (%)	102 (48)	13 (38)	52 (60)	37 (39)	0.19
Malaise: yes, n (%)	62 (29)	14 (41)	30 (35)	18 (19)	0.88
Cough: yes, n (%)	165 (77)	27 (79)	67 (78)	71 (76)	0.61
Appetite loss: yes, n (%)	110 (51)	18 (53)	43 (50)	48 (51)	0.69
Weight loss: yes, n (%)	133 (62)	23 (68)	63 (73)	47 (50)	0.07
Vomiting: yes, n (%)	43 (20)	7 (21)	19 (22)	17 (18)	0.73
BMI, median (IQR)	16 (15–18)	15 (14–17)	15 (14–17)	16 (15–18)	0.26
Hypoxia, median (IQR)	99 (97–100)	98 (97–99)	99 (97–100)	100 (97–100)	0.63
Chest in drawing: yes, n (%)	20 (9)	5 (15)	10 (12)	5 (5)	0.08
Respiratory rate, median (IQR)	34 (31–40)	35 (30–40)	35 (30–40)	34 (32–40)	0.68

### Microbes detected in nasopharyngeal samples

Nucleic acid of at least one of the 33 targeted respiratory microbes was detected in 97 % of 214 NP specimens. The most common bacteria detected were *Moraxella catarrhalis* (64 %), *S. pneumoniae* (42 %), *H. influenzae spp* (29 %) and *Staphylococcus aureus* (22 %) (Table 2). *M. pneumoniae* (9 %), *B. pertussis* (7 %) or *C. pneumoniae* (4 %) were detected less frequently. The most frequently detected viral targets were human metapneumovirus (hMPV) (19 %), rhinovirus (15 %), influenza C virus (9 %), adenovirus (7 %), cytomegalovirus (7 %) and coronavirus O43 (5.6 %) (Table 2).

**Table 2:** Respiratory microbes detected from children presenting with suspected pulmonary tuberculosis^a^

	Total n (%)	^c^TB status n (%)			Unadjasted *p*-value	^#^Adjusted *p*-value
		Definite-TB	Unconfirmed-TB	Unlikely-TB		
Number of participants	214 (100)	34 (16)	86 (40)	94 (44)		
Bacteria						
*M. catarrhalis*	137 (64)	24 (71)	56 (65)	57 (60)	0.64	1.00
*S. pneumoniae*	90 (42)	18 (53)	38 (44)	34 (36)	0.22	1.00
*H. influenzae spp*	62 (29)	8 (24)	25 (29)	29 (31)	0.44	1.00
*S. aureus*	47 (22)	7 (21)	18 (21)	22 (23)	0.79	1.00
*H. influenzae B*	31 (15)	5 (15)	15 (17)	11 (12)	0.97	1.00
*M. pneumoniae*	19 (9)	2 (6)	9 (10)	8 (9)	0.84	1.00
*B. pertussis*	12 (6)	3 (9)	6 (7)	3 (3)	0.43	1.00
*C. pneumoniae*	9 (4)	4 (12)	4 (5)	1 (1)	0.03	0.42
Viruses						
Metapneumovirus A/B	41 (19)	7 (21)	21 (24)	13 (14)	0.60	1.00
Rhinovirus	31 (15)	6 (18)	13 (15)	12 (13)	0.77	1.00
Enterovirus	22 (10)	1 (3)	10 (12)	11 (12)	0.21	1.00
Adenovirus	14 (7)	3 (9)	5 (6)	6 (6)	0.99	1.00
Cytomegalovirus	14 (7)	3 (9)	3 (3)	8 (9)	1.00	1.00
Bocavirus	14 (7)	8 (24)	1 (1)	5 (5)	1.00	1.00
Coronavirus	17 (8)	5 (15)	8 (9)	4 (4)	0.13	0.94
Coronavirus O43	12 (6)	5 (15)	5 (6)	2 (2)	0.02	0.42
Coronavirus HKU	2 (1)	0 (0)	1 (1)	1 (1)	1.00	1.00
Coronavirus NL63	2 (1)	0 (0)	1 (1)	1 (1)	1.00	1.00
Coronavirus 229E	1 (0.5)	0 (0)	1 (1)	0 (0)	na^d^	na^d^
RSV A/B^b^	7 (3)	1 (3)	3 (3)	3 (3)	1.00	1.00
Influenza virus	33 (15)	8 (24)	15 (17)	10 (11)	0.17	0.94
Influenza A	7 (3)	1 (3)	4 (5)	2 (2)	1.00	1.00
Influenza B	7 (3)	1 (3)	2 (2)	4 (4)	1.00	1.00
Influenza C	19 (9)	6 (18)	9 (10)	4 (4)	0.04	0.43
Parainfluenza virus	10 (5)	1 (3)	4 (5)	5 (5)	0.88	1.00
Parainfluenza 1	5 (2)	1 (3)	2 (2)	2 (2)	1.00	1.00
Parainfluenza 2	1 (0.5)	0 (0)	0 (0)	1 (1)	1.00	1.00
Parainfluenza 3	2 (1)	0 (0)	1 (1)	1 (1)	1.00	1.00
Parainfluenza 4	2 (1)	0 (0)	1 (1)	1 (1)	1.00	1.00
Both Bacteria & Viruses	124 (58)	24 (73)	48 (56)	52 (55)	-	0.11
Fungi						
*P. jirovecii*	23 (11)	3 (9)	10 (12)	10 (11)	0.94	1.00

^a^Arranged in order of decreasing colonisation or detection rates

^b^RSV A/B = Respiratory Syncyntial virus A and B

^#^The adjusted *p-*value compensates for multiple comparisons. The *p*-values only compare the presence of each microbe between the definite-TB and unlikely-TB groups. The true proportion of TB in the unconfirmed-TB is unknown hence not included in comparison

^c^TB = Microbiological confirmation of pulmonary Tuberculosis

na^d^ = Non of the children had Coronavirus 229 present, except in the unconfirmed-TB category which is excluded

Seasonal patterns were observed for hMPV, rhinovirus, enterovirus and influenza viruses with peak prevalence in late winter (August) and spring (November). In addition, a seasonal pattern was detected for tuberculosis (Additional file 1: Figure S1). No distinct seasonal patterns were observed for other microbes.

A single bacterial target was detected in 39/214 (18 %) of samples tested. Two bacterial targets were detected in 53/214 (25 %), and three bacterial targets detected in 111/214 (52 %). A single viral target was detected in 71/214 (33 %) of samples, two viral targets in 47/214 (22 %) and three or more viral targets in 10/214 (5 %) specimens. Bacteria alone were detected in 86/214 (40 %) of samples, viruses alone were detected in 11/214 (5 %) of samples and both viruses and bacteria in 117/214 (55 %) of samples. A detailed overview of all possible pairs of co-occuring respiratory microbes, irrespective of TB category (Additional file 1: Table S2). These co-occurences were further tested for significance (Table 3). Significant bacterial-bacterial associations included interactions between *M. catarrhalis* and each of *M. pneumoniae*, *S. pneumoniae* and *H. influenza spp*. Viral-viral associations included: bocavirus and influenza A virus, parainfluenza 1 virus and coronavirus NL63 as well as hMPV and enterovirus. Significant viral - bacterial associations were common particularly between *H. influenza* (type b or non-type b) and a range of viruses, including enteroviruses, hMPV, influenza C, influenza A and cytomegalovirus.

**Table 3:** Summary of significance level for all paired microbe co-occurrences^c^

	Influenza A	Rhinovirus	Influenza B	Parainfuenza 3	Parainfuenza 2	Parainfuenza 4	Coronavirus 29	Coronavirus 63	Coronavirus 43	Coronavirus HK	RSV A/B^a^	Cytomegalovirus	Adenovirus	Enterovirus	Parainfuenza 1	HmPV A/B^b^	*M. pneumoniae*	Bocavirus	*S. aureus*	*C. pneumoniae*	*S. pneumoniae*	*H. influenzae B*	*P. jirovecii*	*M. cartarrhalis*	*Influenza C*	*H. influenzae spp*
Rhinovirus	1																									
Influenza B	1	1																								
Parainfuenza 3	1	0.27	1																							
Parainfuenza 2	1	0.14	1	1																						
Parainfuenza 4	1	0.27	1	1	1																					
Coronavirus 229	1	1	1	1	1	1																				
Coronavirus 63	1	1	1	1	1	1	1																			
Coronavirus 43	1	0.56	0.33	1	1	1	1	1																		
Coronavirus	0.07	1	1	1	1	1	1	1	1																	
RSV A/B^a^	1	0.66	1	1	1	1	1	1	1	1																
Cytomegalovirus	0.07	1	1	1	1	1	0.07	1	0.57	0.12	1															
Adenovirus	0.38	0.90	1	1	1	1	1	1	0.56	0.13	1	0.23														
Enterovirus	1	0.08	0.54	1	1	0.20	1	1	1	1	1	0.80	0.16													
Parainfluenza 1	1	1	1	1	1	1	1	**0.04**	0.25	1	1	1	1	1												
HmPV A/B^b^	0.78	0.57	0.78	0.3	1	1	1	1	0.41	1	1	0.27	0.79	**0.01**	1											
*M. pneumoniae*	0.48	0.29	1	**0.01**	1	0.17	1	1	0.68	1	0.12	1	0.36	0.31	1	0.51										
Bocavirus	**0.02**	0.33	1	1	1	1	1	1	1	0.08	0.24	1	0.42	0.58	1	0.82	1									
*S. aureus*	1	0.72	0.83	1	1	0.39	1	1	0.79	1	0.82	0.37	0.97	0.08	1	0.26	0.83	0.25								
*C. pneumoniae*	1	0.76	1	1	1	1	1	1	1	1	0.26	0.47	0.46	0.63	0.19	1	0.58	0.29	0.89							
*S. pneumoniae*	0.11	0.09	0.62	1	1	0.67	1	1	0.19	0.67	0.98	0.18	0.77	0.63	0.94	**0.03**	0.76	0.73	0.28	0.99						
*H. influenzae B*	1	0.69	1	1	1	0.27	1	1	0.85	1	1	0.63	0.12	**0.02**	1	0.75	**0.04**	1	0.36	0.76	0.43					
*P. jirovecii*	0.55	0.44	0.55	1	1	1	1	0.21	0.38	**0.01**	0.55	0.46	0.81	0.72	1	0.68	0.63	0.60	**0.00**	1	0.70	**0.00**				
*M. cartarrhalis*	0.51	0.07	0.21	0.42	1	0.88	1	0.41	**0.03**	0.87	0.51	0.38	0.97	**0.05**	0.95	0.06	**0.01**	1	0.81	0.94	**0.00**	0.71	0.72			
Influenza C	1	0.79	1	1	1	1	1	1	0.30	1	0.12	1	0.75	0.61	0.06	0.91	**0.02**	0.53	1	0.19	1	**0.00**	0.63	0.45		
*H. influenzae spp*	**0.02**	0.57	0.32	0.49	1	1	1	1	0.47	0.50	0.91	**0.00**	0.39	0.92	0.82	**0.02**	0.69	0.73	0.99	0.52	**0.01**	0.86	0.71	**0.03**	1	
*B. pertussis*	1	0.54	0.33	1	1	1	1	1	0.51	1	1	1	1	0.12	1	0.93	0.29	1	1	0.41	0.94	**0.00**	1	0.13	**0.00**	0.48

^a^RSV A/B = Respiratory Syncyntial virus A and B, ^b^HmPV A/B = Human Metapneumovirus A and B

^c^Each pair of microbe pairs were tested for concurrency and the table reports achieved significance levels (interpreted similar to a *p*-value) irrespective of the TB category. Significant concurrent relationships are highlighted in bold

### Microbial target detection by TB category

**Fig. 1:**
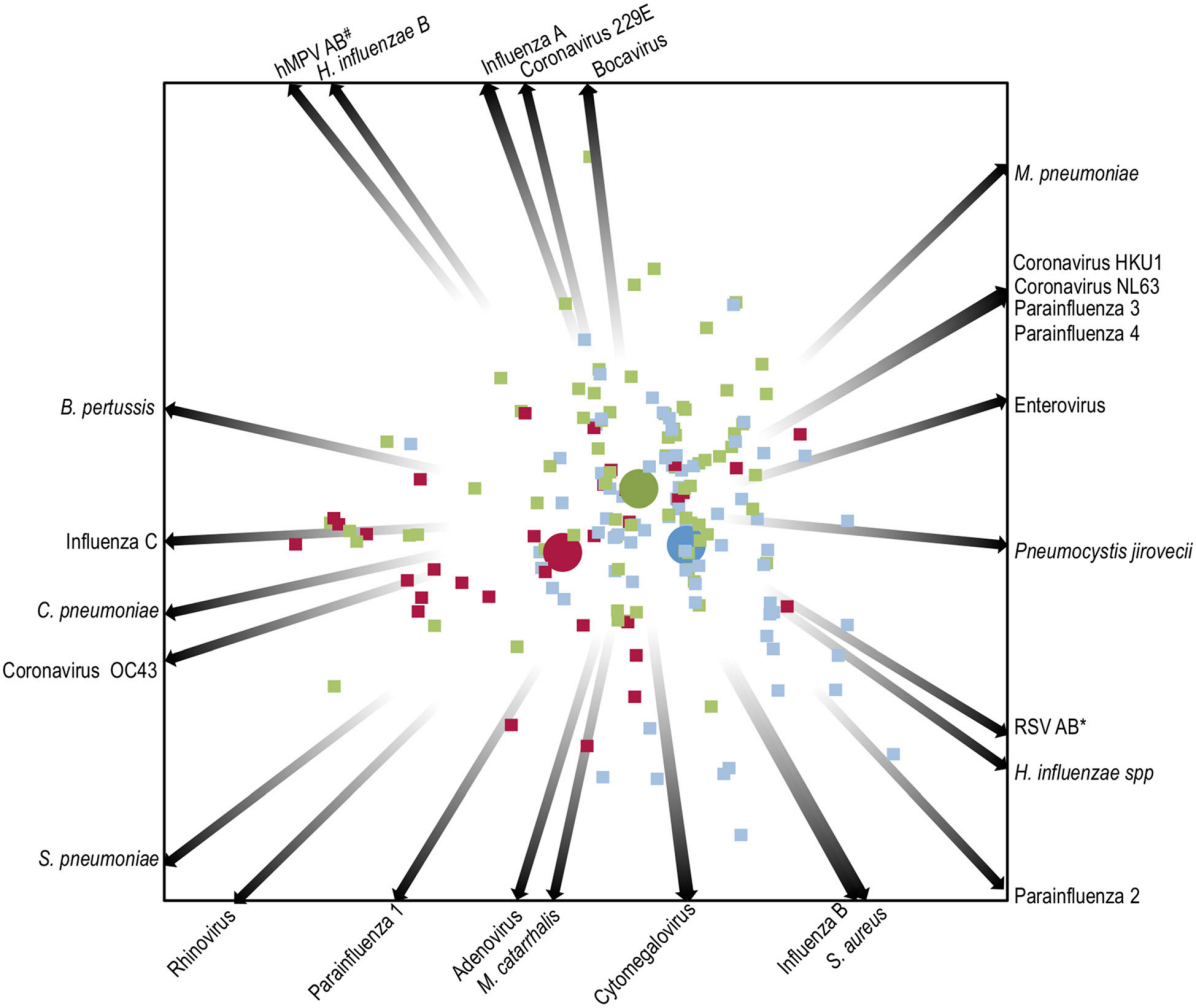
Canonical Variate Analysis (CVA) biplot for graphical visualisation of Linear Discriminant Analysis (LDA). This demonstrates the presence or absence of respiratory microbes in the definite-TB (*red squares*), unconfirmed-TB (*green squares*) and unlikely-TB (*blue squares*) groups

Both bacterial and viral targets were detected in 24/34 (71 %), 52/94 (55 %) and 48/86 (56 %) of NP specimens from children with definite-TB, unconfirmed-TB and unlikely-TB groups, respectively. There were no significant differences in the distribution of individual pathogens (Table 2) by TB category. However, visual inspection of the CVA biplot for graphical visualisation of LDA for all TB categories (Fig. 1) suggests that *C. pneumoniae*, *S. pneumoniae, M. catarrhalis,* coronavirus O43*,* influenza virus C virus, rhinovirus, parainfluenza virus 1, and adenovirus formed the dominant microbial profile in definite-TB cases. In contrast, cytomegalovirus, influenza B, parainfluenza virus 2, RSV A/B, *S. aureus, H. influenzae spp,* and *P. jirovecii* associated with the unlikely-TB group. When the unconfirmed TB group is excluded from the LDA analysis, the LDA biplot is reduced to a one-dimensional plot (Additional file 1: Figure S2). In this case, the presence of rhinovirus, coronavirus 043, adenovirus, parainfluenza 1, hMPV, bocavirus, *C. pneumoniae*, *S. pneumoniae*, *H. influenzae type b, M. catarrhalis,* influenza virus C virus, and *B. pertussis* best discriminated cases with definite-TB from those with unlikely TB.

**Fig. 2:**
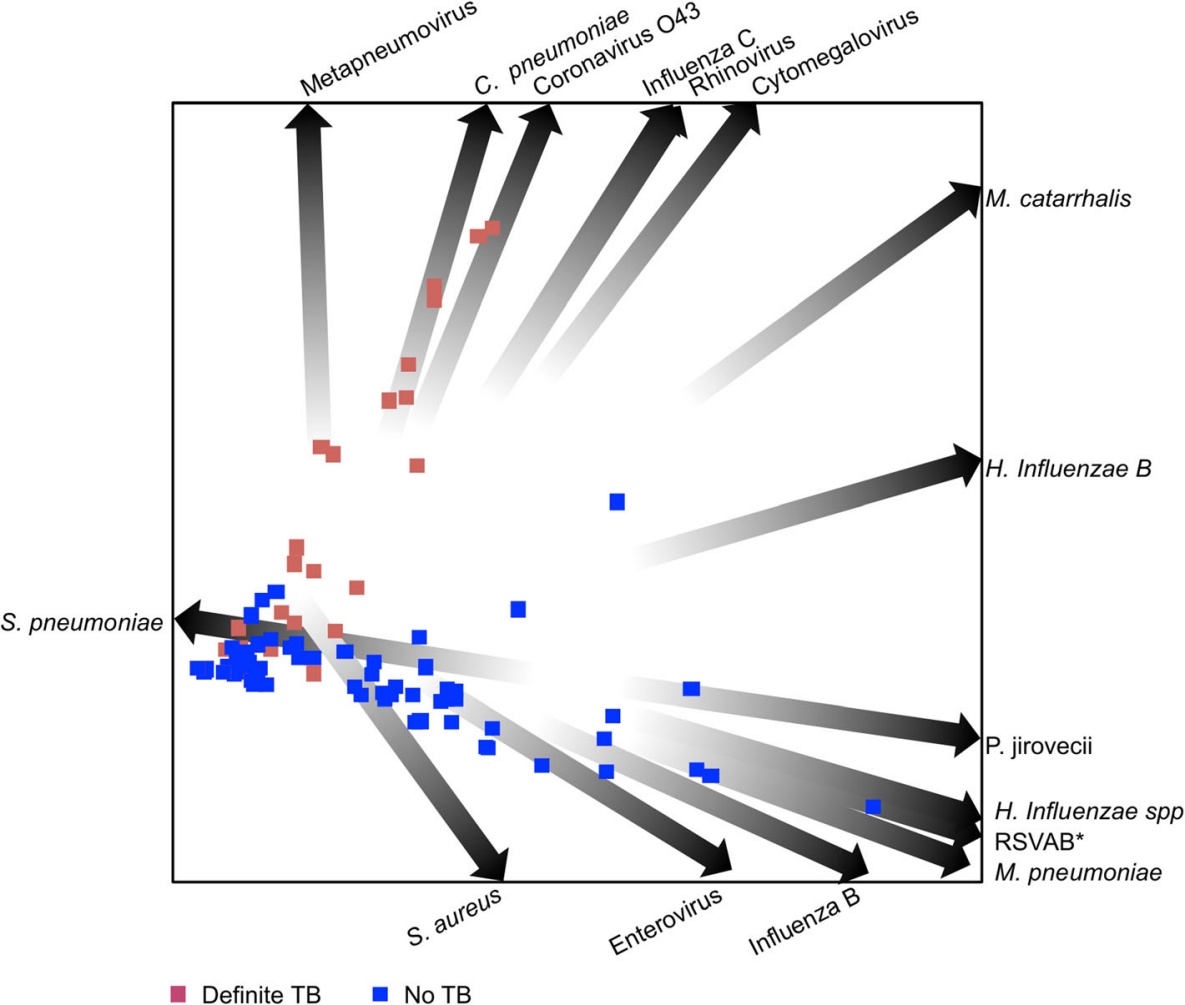
Quadratic Discriminant Analysis (QDA) biplot for graphical visualisation of microbe classification [16]. Clustering of respiratory microbes in the definite-TB (*red squares*) and unlikely-TB (*blue squares*) groups is shown. Arrows indicate the presence of the microbes. *RSVAB = Respiratory Syncytial virus A and B

Quadratic Discriminent Analysis did not identify any significant association between definite-TB and unlikely-TB groups. However, visual inspection of the QDA biplot (Fig. 2) showed that hMPV, coronavirus 043, influenza C virus, rhinovirus, cytomegalovirus and *C. pneumoniae* formed the dominant microbial profile associated with definite-TB cases. In contrast, *M. pneumoniae*, *H. influenzae*, *P. jirovecii*, enterovirus, influenza B virus and RSV A/B were associated with the unlikely-TB category.

## Discussion

This is the first comprehensive detailed study of the diversity of respiratory microbes detected in children presenting with suspected PTB in a TB endemic setting and showed that multiple potential pathogens are present in th nasopharynx of such children.

The FTD33 multiplex real-time PCR detected at least one of the 33 microbial targets in 97 % of NP swabs from children suspected of PTB. Detection of multiple bacterial and viral targets was common. Bacterial species frequently found as commensals in the nasopharynx were most commonly detected [17, 18]. They include *M. catarrhalis* (64 %), *S. pneumoniae* (42 %), *H. influenzae spp* (29 %), and *S. aureus* (22 %). In addition, potential pathogenic organisms were detected in the nasopharynx including RSV, *C. pneumoniae* and *B. pertussis.*

The prevalence of *B. pertussis* was 6 % in our study. Similar detection rates (1–9 %) were reported in other South African settings in children with LRTI including at our study site, 10–20 years post transition from whole-cell vaccines to acellular vaccines (South African infants are vaccinated with DTaP-IPV/HIB; Pentaxime®, Sanofi Pasteur) [19].

The prevalence of some clinically relevant viral targets (RSV, bocavirus and adenovirus) in this cohort is lower than that previously reported in children with LRTI [20–22]. The observed differences may be explained by our enrolment criteria which targeted symptoms suggestive of PTB. However, viral PCR positivity of hMPV, enterovirus and influenza virus is similar to a recent case-control study that also showed their association with community acquired pneumonia [20]. As with bacteria, care needs to be taken with the interpretation of molecular detection of some viruses in NP specimens, since target nucleic acid may be detected for some time after resolution of symptoms, and from otherwise healthy children [23, 24].

In this study, some microbes showed no association with any of the TB categories. These included *M. catarrhalis*, and *S. pneumoniae*. A randomised controlled trial of the efficacy of PCV9 in South African children showed decreased rates of culture-confirmed and clinically diagnosed TB in PCV9 recipients hospitalised with LRTI compared with placebo recipients (relative risk reduction 43 %) [6]. This suggests that coinfection with *M. tuberculosis* and *S. pneumoniae* causes severe infection requiring hospitalization. Although common in our cohort, *S. pneumoniae* did not cluster together with the TB or unlikely-TB groups, however we measured NP colonization which is likely to be an inaccurate measure for the contribution of *S. pneumoniae* to LRTI.

We have recently shown [5] that specific pathogens (specifically *B. pertussis*, influenza virus, RSV, adenovirus, parainfluenzavirus, bocavirus) are detected significantly more frequently from the NP of children with pneumonia than age-matched controls. In this study we detected all of these organisms, irrespective of TB-classification, suggesting that these pathogens may play a role in the exacerbation of symptoms in children with TB as well as accounting for the respiratory illness of a subset of the children without TB.

Whilst we detected multiple significant co-occurrences between different microbes in this study, these require more detailed assessment in a larger group of children. For example, the co-occurrence between *M. catarrhalis, S. pneumoniae* and *H. influenzae,* may reflect age-specific colonization patterns as previously reported [25, 26]. Other microbial co-occurrences, such as the association between *S. aureus* and *P. jorovecii,* were unexpected. We are currently conducting a larger, longitudinal study to better understand these co-occurences [27].

We were unable to identify significant associations between individual nasopharyngeal microbes and TB classification. Discriminant analysis identified that the presence of *C. pneumoniae*, hMPV, coronavirus O43, influenza C virus, rhinovirus and cytomegalovirus best discriminated children with definite TB. The significance of co-detection of these microbes in children with TB is unclear, and needs to be further assessed. One possibility is that the relative immune suppression or lung pathology associated with PTB may render the host susceptible to other respiratory infections, or alternatively, that intercurrent infection may be immunosuppressive, predisposing to an accelerated clinical course or likelihood of symptoms in children with PTB. Active TB is associated with suppression of cellular immune responses, which are critical for the control of intracellular infections [28] such as many of those associated with definite TB in this study. However, in this study, individual microbes were each only detected in small numbers of children which limits our ability to draw firm conclusions in this regard.

A recent South African study has shown an increased risk of death in adults with TB-influenza A virus co-infection (adjusted relative risk ratio [aRRR] 6.1) compared to TB infection alone [29]. In contrast, de Paus et al. did not find a correlation between the seroprevalence of influenza antibodies and the development of clinically active TB in an Indonesian cohort [30]. They did however show an association between elevated antibody titres against influenza A and the clinical stage of TB lung disease suggesting recent re-infection with influenza precedes clinical presentation with PTB [30].

A limitation of this study is the lack of a control group of children without lower respiratory symptoms. We are therefore unable to infer whether the pathogens detected played a role in the development or exacerbation of symptoms in this cohort. Further limitations include sampling of the nasopharynx rather than the lower respiratory tract, limiting the ability to infer causality for lung co-pathogens. *Klebsiella pneumoniae*, *Legionella spp* and *Salmonella* targets were excluded from analysis due to problems with assay specificity for these targets.

## Conclusion

In conclusion, this study describes the detection of multiple respiratory microbes in the nasopharynx of children hospitalised with suspected PTB. Whilst there was no clear separation between the pathogens present in the airways of children with and without PTB, *C. pneumoniae*, hMPV, coronavirus O43, influenza C virus, rhinovirus and cytomegalovirus formed the dominant microbial profile in children with PTB but this failed to reach statistical significance on testing of each individual microbe. In contrast, *P. jirovecii*, *H. influenzae spp*, RSV, *M. pneumoniae*, influenza B virus and enteroviruses were more consistently detected in children without TB although not statistically significant. This pilot work may signal broader differences in the microbial ecology of the upper respiratory tract of these children, which warrants further study.

## Additional file

Additional file 1: Figure S1. Seasonal distribution of viruses and bacteria. **Figure S2.** Canonical Variate Analysis (CVA) biplot depicting the spread of respiratory pathogens in the definite TB (red line) and not TB (blue line) groups only. Observations under each group are denoted by “+” signs and the median of each group by the red and blue ovals. **Table S1.** Target pathogens in the FTD respiratory pathogens 33 multiplex realtime PCR assay. **Table S2.** Summary of all paired pathogen co-occurrence counts *. **Table S3.** Risk factors associated with the occurrence of each microbes. (PDF 565 kb)
