# Supplementary material for: Respiratory microbes present in the nasopharynx of children hospitalised with suspected pulmonary tuberculosis in Cape Town, South Africa
Source: BMC Infect Dis. 2016 Oct 24;16:597. doi: 10.1186/s12879-016-1934-z (PMC5075757; doi:10.1186/s12879-016-1934-z)
Supplement: Additional file 1: Figure S1. — Seasonal distribution of viruses and bacteria. Figure S2. Canonical Variate Analysis (CVA) biplot depicting the spread of respiratory pathogens in the definite TB (red line) and not TB (blue line) groups only. Observations under each group are denoted by “+” signs and the median of each group by the red and blue ovals. Table S1. Target pathogens in the FTD respiratory pathogens 33 multiplex realtime PCR assay. Table S2. Summary of all paired pathogen co-occurrence counts *. Table S3. Risk factors associated with the occurrence of each microbes. (PDF 565 kb) [file 12879_2016_1934_MOESM1_ESM.pdf]

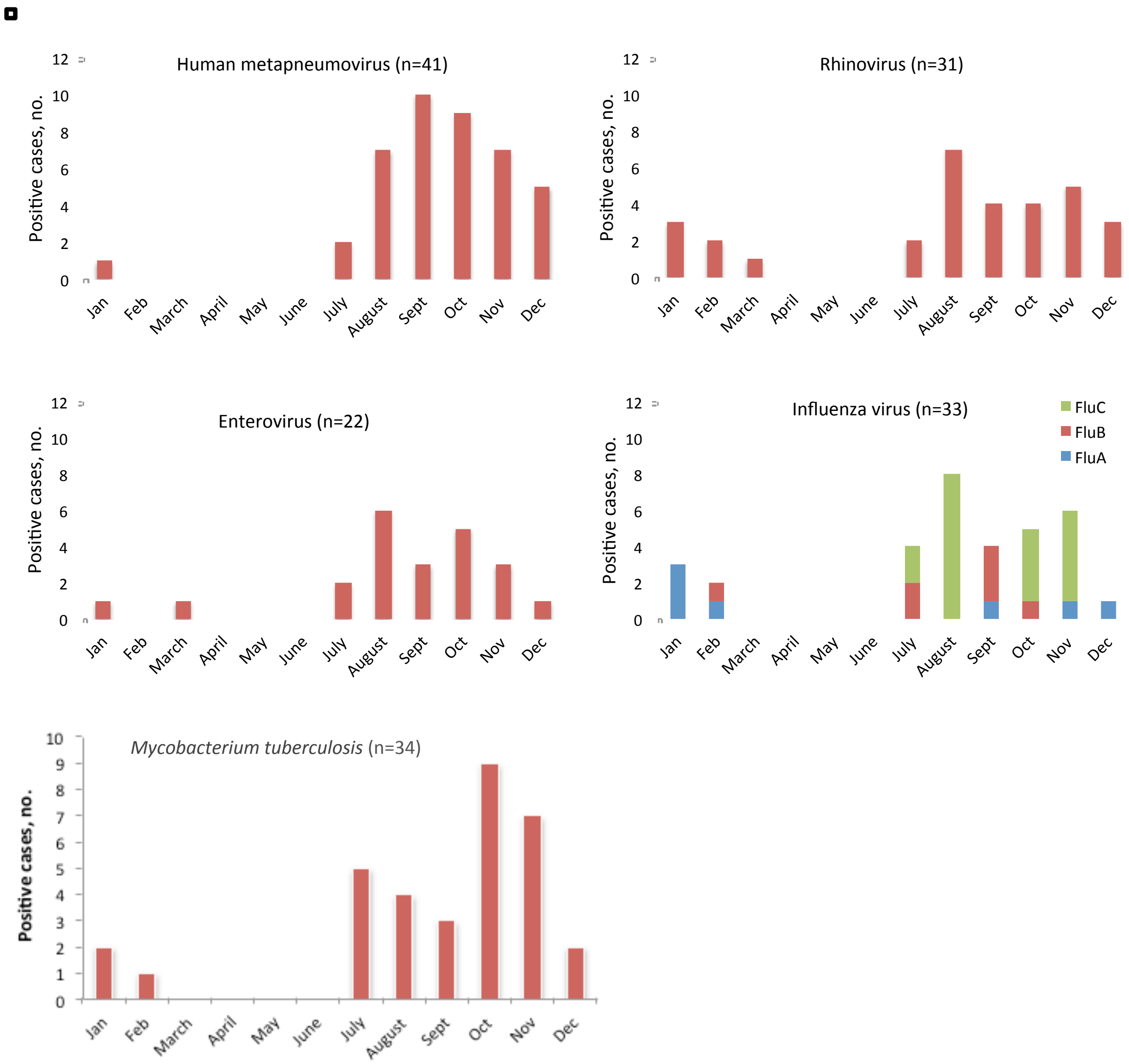

Figure S1: Seasonal distribution of viruses and bacteria.

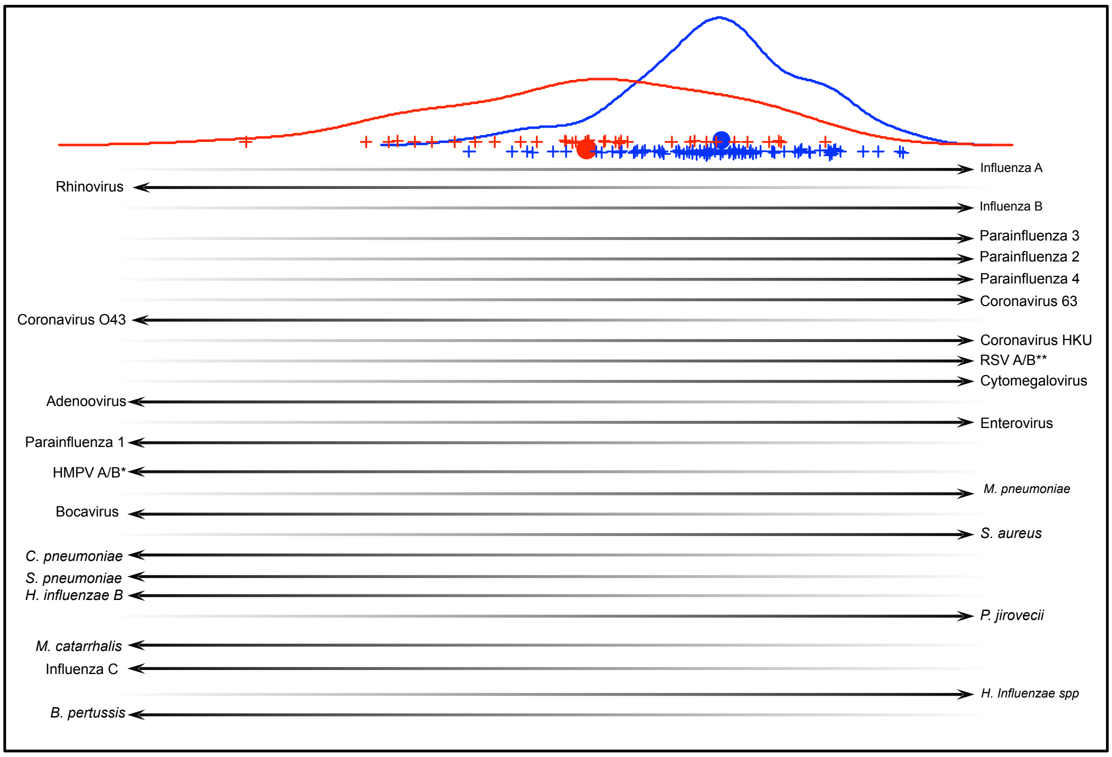

Supplementary figure 2: Canonical Variate Analysis (CVA) biplot depicting the spread of respiratory pathogens in the definite TB (red line) and not TB (blue line) groups only. Observations under each group are denoted by "+" signs and the median of each group by the red and blue ovals.

Table S1: Target pathogens in the FTD respiratory pathogens 33 multiplex real-time PCR assay.

[a]Mplx No.	[b]Primer/ Probe mix	Target pathogen (Nucleic acid type)	Target sequence genes
1	FluAB-RH	Influenza A (**RNA**)	Matrix gene (*pos*1)
		Influenza B (**RNA**)	Segment 8 NS1/NEP
		Rhinovirus (**RNA**)	5'untranslated region (5' UTR)
2	Para-EAV	Parainfluenza 3 (**RNA**)	Hemagglutinin-neuraminidase (HN) mRNA
		Parainfluenza 2 (**RNA**)	Hemagglutinin-neuraminidase (HN) mRNA
		Parainfluenza 4 (**RNA**)	Hemagglutinin-neuraminidase (HN) mRNA
		Internal control*	
3	Cor	Coronavirus 229 (**RNA**)	Nucleocapsid protein (N) gene
		Coronavirus 63 (**RNA**)	Nucleocapsid protein (N) gene
		Coronavirus HKU1 (**RNA**)	Nucleocapsid protein (N) gene
		Coronavirus 43 (**RNA**)	Nucleocapsid protein (N) gene
4	BoMpPf1	Parainfluenza 1 (**RNA**)	Hemagglutinin-neuraminidase (HN) mRNA
		Human Metapneumoviruses A/B (**RNA**)	Fusion glycoprotein (F) gene
		Bocavirus (**DNA**)	Nonstructural protein (NP1) gene
		Mycoplasma pneumoniae (**DNA**)	Adhesin P1
5	RsEPAcmv	Respiratory syncytial viruses A/B (**RNA**)	Nucleocapsid protein (N) gene
		Cytomegalovirus (**DNA**)	US7+US8 genes
		Enterovirus (**RNA**)	Parts of domain IV and V
		Parechovirus (**RNA**)	5'untranslated region (UTR)
		Adenovirus (**DNA**)	Hexon gene
6	Bac	Staphylococcus aureus (**DNA**)	Sensor histidine kinase (*vicK*) gene
		Chlamydophila pneumoniae (**DNA**)	RNA polymerase beta chain gene
		Haemophilus influenza B (**DNA**)	capsular export (*BexA*) gene
		Streptococcus pneumoniae (**DNA**)	LytA gene
7	KLePSa	Pneumocystis jirovecii (**DNA**)	(mtLSU) rRNA gene
		Legionella spp (**DNA**)	16 S rRNA
		Klebsiella pneumoniae (**DNA**)	Hemolysin (*Khe*) gene
		Salmonella spp (**DNA**)	Tetrathionate subunit B (ttrB)
8	MoBoCH	Moraxella catarrhalis (**DNA**)	Outer membrane proteins (*cop*B) gene
		Influenza C (RNA)	Matrix gene
		Bordetella pertussis (**DNA**)	Insertion sequence (IS481)
		Haemophilus influenza spp (**DNA**)	Outer membrane proteins *(ompP2)* gene

Note. *Equine arteritis virus (EAV) based Internal control. [a]Mplx No.= Multiplex PCR reaction number as indicated in the FTD resp33 kit. [b]Pathogen names as indicated on package inserts for each multiplex reaction mix.

Table S2: Summary of all paired pathogen co-occurrence counts *

	Influenza A	Rhinovirus	Influenza B	Parainfuenza 3	Parainfuenza 2	Parainfuenza 4	Coronavirus 29	Coronavirus 63	Coronavirus 43	Coronavirus HK	RSV A/B[a]	Cytomegalovirus	Adenovirus	Enterovirus	Parainfuenza 1	HmPV A/B[b]	*M. pneumoniae*	Bocavirus	*S. aureus*	*C. pneumoniae*	*S. pneumoniae*	*H. influenzae B*	*P. jirovecii*	*M. cartarrhalis*	*Influenza C*	*H. influenzae* sp	*B. pertussis*
Influenza B	**7**																										
Rhinovirus	0	**31**																									
Influenza B	0	0	**7**																								
Parainfuenza 3	0	1	0	**2**																							
Parainfuenza 2	0	1	0	0	**1**																						
Parainfuenza 4	0	1	0	0	0	**2**																					
Coronavirus 229	0	0	0	0	0	0	**1**																				
Coronavirus 63	0	0	0	0	0	0	0	**2**																			
Coronavirus 43	0	2	1	0	0	0	0	0	**12**																		
Coronavirus	1	0	0	0	0	0	0	0	0	**2**																	
RSV A/B[a]	0	1	0	0	0	0	0	0	0	0	**7**																
Cytomegalovirus	2	0	0	0	0	0	1	0	1	1	0	**14**															
Adenovirus	1	1	0	0	0	0	0	0	1	1	0	2	**14**														
Enterovirus	0	6	1	0	0	1	0	0	0	0	0	1	3	**22**													
Parainfluenza 1	0	0	0	0	0	0	0	1	1	0	0	0	0	0	**5**												
HmPV A/B[b]	1	6	1	1	0	0	0	0	3	0	0	4	2	9	0	**41**											
M. pneumoniae	1	4	0	2	0	1	0	0	1	0	2	0	2	3	0	4	**19**										
Bocavirus	2	2	0	0	0	0	0	0	0	1	1	0	1	1	0	1	0	**8**									
S. aureus	0	6	1	0	0	1	0	0	2	0	1	4	1	8	0	11	3	3	**47**								
C. pneumoniae	0	1	0	0	0	0	0	0	0	0	1	1	1	1	1	0	1	1	1	**9**							
S. pneumoniae	5	17	3	0	0	1	0	0	7	1	1	8	5	9	1	23	7	3	22	1	**90**						
H. influenzae B	0	4	0	0	0	1	0	0	1	0	0	2	4	7	0	5	6	0	8	1	14	**31**					
P. jirovecii	1	4	1	0	0	0	0	1	2	2	1	2	1	2	0	4	2	1	**13**	0	9	9	**23**				
M. cartarrhalis	5	24	6	2	0	1	0	2	11	1	5	10	6	18	2	31	17	2	28	4	75	19	14	**137**			
Adenovirus	0	2	0	0	0	0	0	0	2	0	2	0	1	2	2	2	5	1	0	2	3	8	2	13	**19**		
H. influenzae spp	5	9	3	1	0	0	0	0	4	1	1	9	5	4	1	18	5	2	8	3	35	7	6	46	0	**62**	
B. pertussis	0	2	1	0	0	0	0	0	1	0	0	0	0	3	0	1	2	0	0	1	3	7	0	10	7	4	**12**

**The pathogen abbreviation names in the top row are derived from the first column. The diagonal line represents the total count of each pathogen detected. [a]RSV A/B = Respiratory Syncytial virus A and B,* [b]HmPV A/B = Human Metapneumovirus A and B

Table S3: Risk factors associated with the occurrence of each microbes.

	Age	Gender	Smoking	Night sweats	Fever	Malaise	Cough	Appetite loss	Weight loss	Vomiting	BMI	**Hypoxia**	**Chest in-drawing**	**Respiratory rate**	HIV infection
**Bacteria**															
M. catarrhalis	1	1	1	1	1	1	1	1	1	1	1	1	1	1	1
S. pneumoniae	1	1	1	1	1	1	1	1	1	1	1	1	1	1	1
H. influenzae spp	1	1	1	1	1	1	1	1	1	1	1	1	1	1	1
S. aureus	1	1	1	1	1	1	1	1	1	1	1	1	1	1	1
H. influenzae B	1	1	1	1	1	1	1	1	1	1	1	1	1	1	1
M. pneumoniae	1	1	1	1	1	1	1	1	1	1	1	1	1	1	1
B. pertussis	1	1	1	1	1	1	1	1	1	1	1	1	1	1	1
C. pneumoniae	1	1	1	1	1	1	1	1	1	1	1	1	1	1	1
**Viruses**															
Metapneumovirus A/B	1	1	1	1	1	1	1	1	1	1	1	1	1	1	1
Rhinovirus	1	1	1	1	1	1	1	1	1	1	1	1	1	1	1
Enterovirus	1	1	1	1	1	1	1	1	1	1	1	1	1	1	1
Adenovirus	1	1	1	1	1	1	1	1	1	1	1	1	1	1	1
Cytomegalovirus	1	1	1	1	1	1	1	1	1	1	1	1	1	1	1
Bocavirus	1	1	1	1	1	1	1	1	1	1	1	1	1	1	1
Coronavirus	1	1	1	1	1	1	1	1	1	1	1	1	1	1	1
Coronavirus O43	1	1	1	1	1	1	1	1	1	1	1	1	1	1	1
Coronavirus HKU	1	1	1	1	1	1	1	1	1	1	1	1	1	1	1
Coronavirus NL63	1	1	1	1	1	1	1	1	**0.46**	1	1	1	1	1	1
Coronavirus 229E	1	1	1	1	1	1	1	1	1	1	1	1	1	1	1
RSV A/B*	1	1	1	1	1	1	1	1	1	1	1	1	1	1	1
Influenza virus	1	1	1	1	1	1	1	1	1	1	1	1	1	1	1
Influenza A	1	1	1	1	1	1	1	1	1	1	**0.09**	1	1	1	1
Influenza B	1	1	1	1	1	1	1	1	1	1	1	1	1	1	1
Influenza C	1	1	1	1	1	1	1	1	1	1	1	1	1	1	1
Parainfluenza virus	1	1	1	1	1	1	1	1	1	1	1	1	1	1	1
Parainfluenza 1	1	1	1	1	1	1	1	1	1	1	1	1	1	1	1
Parainfluenza 2	1	1	1	1	1	1	1	1	1	1	1	1	1	1	1
Parainfluenza 3	1	1	1	1	1	1	1	1	1	1	1	1	1	1	1
Parainfluenza 4	1	1	1	1	1	1	1	1	1	1	1	**0.48**	1	1	1
**Fungi**															
P. jirovecii	1	1	1	1	1	1	1	1	1	1	1	1	1	1	1

Logistic regression performed of all risk factors on each pathogen. p-values corrected for simultaneous testing with Benjamini Hochberg. No statistically significant effects found at 5% level.
